# Tuning CARs: recent advances in modulating chimeric antigen receptor (CAR) T cell activity for improved safety, efficacy, and flexibility

**DOI:** 10.1186/s12967-023-04041-6

**Published:** 2023-03-15

**Authors:** Piotr Celichowski, Marcello Turi, Sandra Charvátová, Dhwani Radhakrishnan, Neda Feizi, Zuzana Chyra, Michal Šimíček, Tomáš Jelínek, Juli Rodriguez Bago, Roman Hájek, Matouš Hrdinka

**Affiliations:** 1 Department of Haematooncology, Faculty of Medicine, University of Ostrava, Ostrava, Czech Republic; 2 Department of Haematooncology, University Hospital Ostrava, Ostrava, Czech Republic; 3 Faculty of Science, University of Ostrava, Ostrava, Czech Republic; 4 Department of Internal Clinical Sciences, Anesthesiology and Cardiovascular Sciences, Sapienza University of Rome, Rome, Italy

**Keywords:** Chimeric antigen receptor, CAR, Cancer, Immunotherapy, T cell, Synthetic, Regulation, Cell therapy

## Abstract

Cancer immunotherapies utilizing genetically engineered T cells have emerged as powerful personalized therapeutic agents showing dramatic preclinical and clinical results, particularly in hematological malignancies. Ectopically expressed chimeric antigen receptors (CARs) reprogram immune cells to target and eliminate cancer. However, CAR T cell therapy's success depends on the balance between effective anti-tumor activity and minimizing harmful side effects. To improve CAR T cell therapy outcomes and mitigate associated toxicities, scientists from different fields are cooperating in developing next-generation products using the latest molecular cell biology and synthetic biology tools and technologies. The immunotherapy field is rapidly evolving, with new approaches and strategies being reported at a fast pace. This comprehensive literature review aims to provide an up-to-date overview of the latest developments in controlling CAR T cell activity for improved safety, efficacy, and flexibility.

## Background

Over the last decade, therapies with genetically engineered immune cells such as chimeric antigen receptors (CAR) T cells and T cell receptor (TCR) modified T cells have revolutionized personalized cancer treatment. With several products already approved for clinical use and hundreds of products in preclinical development and clinical testing, immunotherapies based on genetically engineered autologous CAR T cells specific for tumor-associated antigens are gaining growing attention from scientists and clinicians, the pharma industry, and the public.

The main limitations of current autologous CAR T cell products include high cost and manufacturing time (availability), cancer resistance or evasion leading to relapse and therapy failure (efficacy), and off-target side effects and toxicities (safety). As true living drugs, CAR T cells require special considerations for their formulation, administration, biodistribution, persistence, and mitigation of potential side effects. The first safety concern arises shortly after infusion when CAR T cells engage with their target antigens and expand in response by 100- to 10 000-fold [1, 2]. Unfortunately, the exact magnitude of CAR T cell reactivity is highly individualized. It can result in adverse reactions such as inflammatory cytokine release syndrome (CRS), tumor lysis syndrome, immune effector cell-associated neurotoxicity syndrome, off-target toxicity or cytopenia [3–12].

It is important to note that the activity of CAR proteins can differ depending on the overall construct design. The selection of signaling domains, such as immunoreceptor tyrosine-based activation motifs (ITAMs), incorporated into the CAR protein [13–15], as well as the affinity and engaging kinetics of the particular antigen-binding domain [16], can significantly affect the outcome of the therapy. Thus, predicting the effectiveness of CAR T cell therapy infusion can be difficult, as it could vary for each CAR construct.

Due to all that reasons, adverse effects can be challenging to manage and pose risks during treatment. Therefore, precise control over infused CAR T cell activation, expansion, and persistence is highly desirable from a clinical perspective. To address these challenges, scientists are implementing various ingenious molecular cell biology and synthetic biology methods and technologies, which are systematically discussed in the respective parts of this review.

**Fig. 1:**
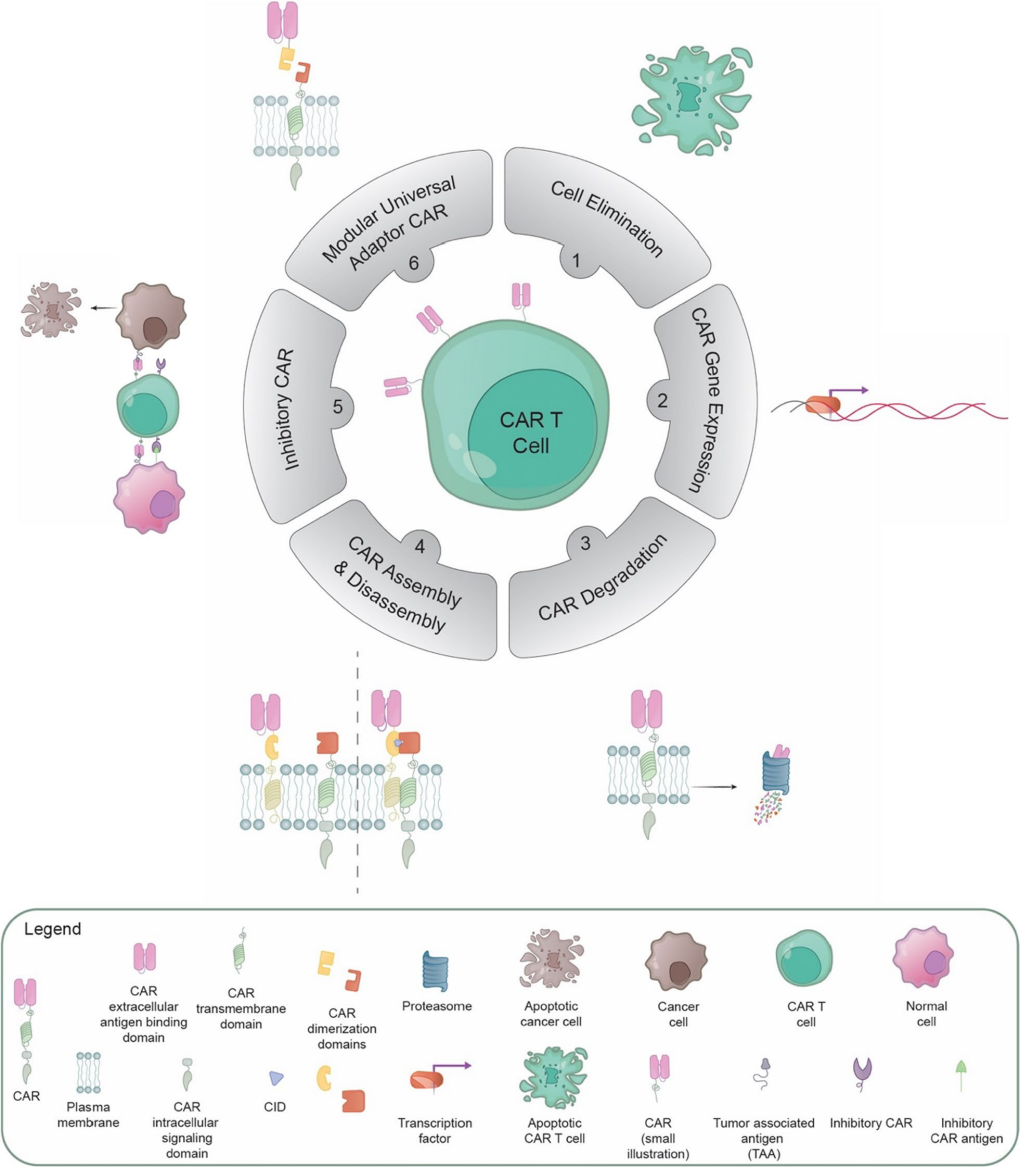
Schematic overview of the various approaches developed to modulate CAR T cell activities as discussed in this review. Six broad categories are discussed: (1) Elimination of therapeutic cells, (2) Regulation of CAR gene expression, (3) Inducible CAR protein degradation, (4) Inducible formation of a functional CAR or inducible CAR disassembly, (5) Inhibitory CARs, and (6) Modular universal adaptor CAR platforms. CID, chemical inducer of dimerization

Many excellent reviews have been published on emerging CAR T cell technologies. The reviews cover a large variety of topics, including CAR switches and modular approaches [17–25], approaches to side effect management [14, 15, 26, 27], innovative design of the CARs [28–34], CAR T cell modifications for targeting to solid tumors [25, 35, 36], or general reviews about the broad topic [14, 15, 37–43]. However, a comprehensive description of experimentally tested approaches to control CAR T cell functions might provide a valuable reference to researchers and clinicians. This review explores the latest trends and technologies in CAR T cell-based immunotherapies that provide more control over CAR-expressing cells (Fig. 1). The primary focus will be on conventional CAR T cell subsets; however, most of the presented technologies can also be applied to other T cell subsets, Natural Killer (NK) cells, and other cell types.

### Elimination of therapeutic cells

The suicide genes, molecular safety switches, and inhibitory corticosteroids represent the earliest and still most popular rational approaches to managing unpredictable CAR T cell expansion and toxicities in patients. Essentially, a safety switch is an ectopically expressed safeguard gene, which can be activated on demand to remove the infused therapeutic cells from the body rapidly and permanently, in case of an adverse immune reaction [23, 24, 44–46]. An ideal safety switch should be capable of depleting the transferred T cells with minimal injury to normal tissues.

**Fig. 2:**
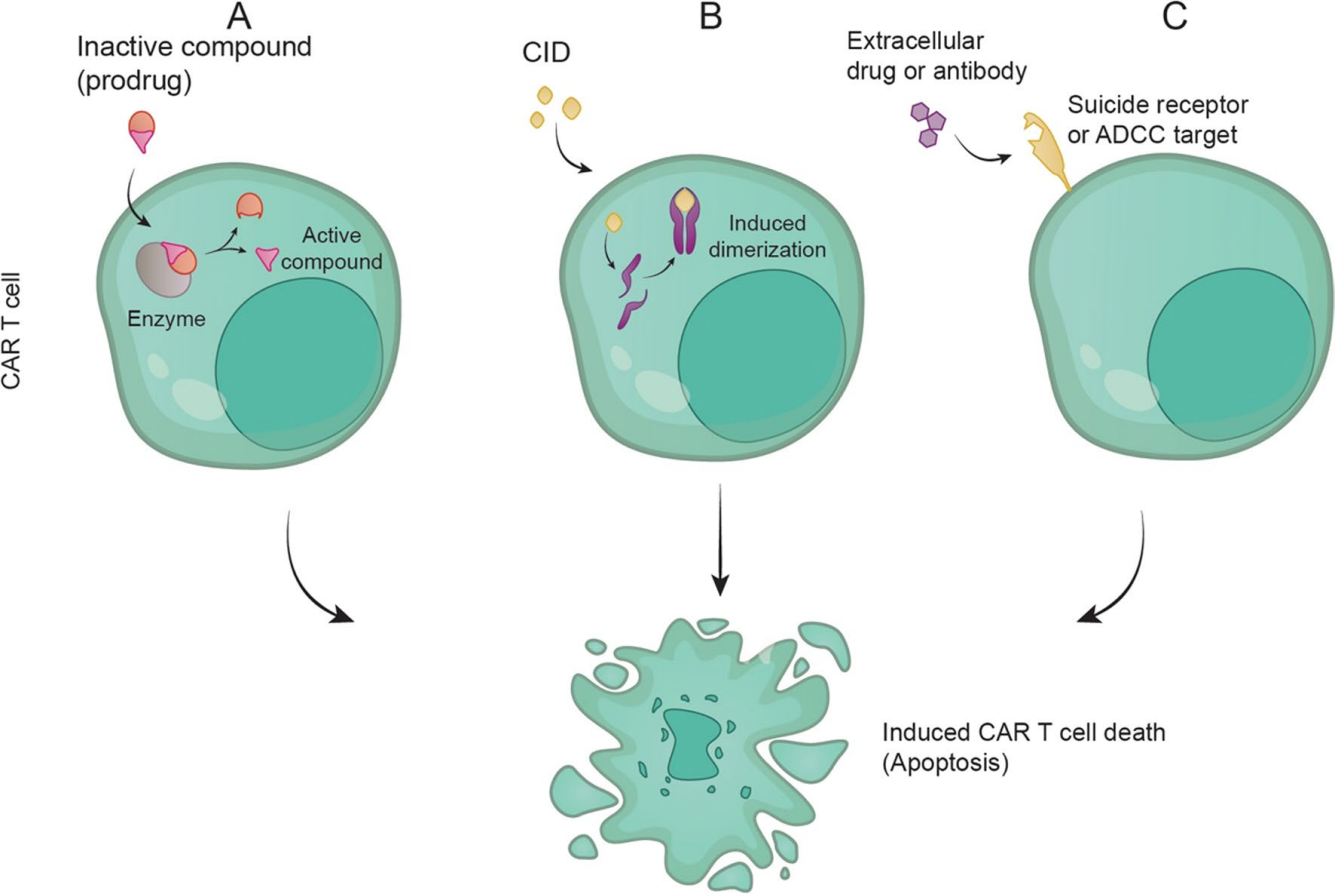
The action of suicide genes. **A** Internal suicide gene is an enzyme that changes a non-toxic drug to a toxic compound that triggers apoptosis. **B** Dimerization with a small drug that causes activation of apoptosis-triggering protein. CID, chemical inducer of dimerization. **C** Surface suicide receptor or ADCC target triggering apoptosis upon contact with an extracellular drug or antibody

The cell elimination process exploits natural cell death mechanisms such as activating intrinsic or extrinsic apoptotic pathways or complement- or antibody-dependent cell-mediated cytotoxicity (ADCC) with subsequent clearance of cellular remains by phagocytic cells [47–58]. A simplified overview of those mechanisms is presented in Fig. 2. As such, suicide genes represent the ultimate safety measures, however, at the expense of permanent loss of the therapeutic cell population and desired tumor killing. Various exogenously administered compounds can activate a specific suicide gene and execute cell killing [49–53, 57, 59–61]. In the following paragraphs, the most common safety strategies will be presented as well as a discussion about their utility in CAR T cell-based therapies.

#### Herpes simplex virus thymidine kinase (HSV-TK)

One of the first-in-class and best-characterized suicide genes exploits the herpes simplex virus I–derived thymidine kinase gene. The system is based on the ability of HSV-TK to phosphorylate nucleoside analogs, such as acyclovir or ganciclovir (GCV), to nucleoside monophosphate, which is subsequently phosphorylated into the nucleoside triphosphate. When nucleoside triphosphate analogs incorporate into newly synthesized DNA strands, it potently inhibits DNA synthesis and triggers mechanisms leading to cell death (Fig. 2A) [50, 51]. The HSV-TK system proved safe and effective in studies with therapeutic T cells and even in phase I–II clinical trials [62–65]. However, other studies report severe limitations of the HSV-TK system, such as the incomplete killing of modified T cells, the requirement for multiple doses of GCV over several days, and high HSV-TK immunogenicity with subsequent elimination of HSV-TK-expressing cells [47, 48, 62, 65, 66].

Moreover, the HSV-TK suicide system also has potential clinical incompatibility leading to unwanted elimination of modified T cells in the case of cytomegalovirus infection treatment with GCV [67, 68]. Continuous development of improved HSV-TK variants resulted in the codon-optimized A168H mutant TK-HSV (TK.007), which demonstrated the highest efficacy, however, at the cost of increased bystander effect [69]. So far, a broad clinical application of the HSV-TK system in CAR cell therapies remains questionable, and most likely, HSV-TK will be outperformed by other suicide gene systems with fewer drawbacks.

#### Inducible caspase 9 (iC9)

Another commonly used suicide gene system utilizes the modified human protein Caspase-9, a key ubiquitously expressed mediator of the extrinsic apoptotic pathway [49, 70]. In this pathway, oligomerization of Caspase-9 in response to extracellular proapoptotic stimuli is a critical step in Caspase-9 activation and triggering the apoptotic cascade. The inducibility of the caspase-9 suicide gene system is achieved by fusing Caspase-9 with the homo- or heterodimerization domains present in FK506-binding proteins (FKBPs). These oligomerization domains can be effectively crosslinked by a small-molecule chemical inducer of dimerization (CID) agents such as FK1012, FK506, rapamycin, AP20187, AP1903 (Rimiducid), and others (Fig. 2B) [47, 61].

Several modifications recently improved the flexibility of the iC9 system. For instance, an alternative method of Cas9 activation based on Rimiducid-inducible MyD88 and CD40 signaling elements can offset potential toxicity risks by an orthogonally regulated, rapamycin-induced, caspase-9-based safety switch [71, 72]. In vitro, the iC9 system achieves almost complete clearance of transduced human cells [73]. However, the system was also successfully and safely used in animal models [71, 74–76] and in clinical settings [77–80]. For example, upon administering a single dose of AP1903 to patients who developed graft-versus-host disease (GvHD) in response to T cell therapy, iC9-induced apoptosis rapidly eliminated more than 85–90% of the iC9 expressing T cells from circulation [78, 80]. Similarly, in a mouse preclinical model, the pharmacologic activation of the iC9 safety switch achieved up to 90% reduction in peripheral blood CAR T cells preferentially killing activated cells expressing high levels of the transgene [77, 79]. The observed dose-dependent killing of iC9 cells induced by dimerizing drugs can be further exploited to limit CAR T cell expansion during CRS with lower doses or ablate CAR T-cell completely with higher doses to restore normal B cell populations [76]. Compared to HSV-TK, no immunogenicity or in vivo toxicities of iCas9-based suicide switches have been reported. Recently, the iC9 system has been gaining popularity as more studies highlight the potential of iC9 as an excellent candidate for a broad application in cell-based therapies and high safety in clinical studies [44, 81].

#### Fas-associated death domain-containing protein (FADD)

Like Caspase-9, FADD is a crucial component of the cellular apoptotic pathways, and a suicide switch system analogous to iC9 was developed to activate FADD fused to heterodimerization domains and trigger apoptosis (Fig. 2B). This system shows high killing efficiency (up to 90%) in transduced T cells after administration of the dimerizing drug [52, 82–85]. While these results look promising, the FADD system is not as popular as other suicide genes, and more evaluations in clinical settings are needed for broader adoption.

#### CD20/Rituximab

The detection and selection of therapeutic cells modified with intracellular suicide switches require co-expression with the CAR or another marker gene, which is inconvenient for most applications. Therefore, alternative strategies utilizing cell surface elimination markers simplify and streamline cell engineering processes. The most common CD20-based safety switch takes advantage of human (therefore non-immunogenic) protein CD20 and chimeric monoclonal CD20 antibodies, such as the FDA-approved antibody Rituximab, to kill target cells in the ADCC process mediated by NK cells (Fig. 2C) [53, 60]. For example, combining two CD20 mimotopes with a CD34 epitope (called RQR8) and a CD8-derived membrane anchor resulted in a chimeric surface protein that allows for selective cell sorting and on-demand cell depletion with Rituximab [86]. Furthermore, a CD20-based safety switch might be combined with a CAR to create an all-in-one CAR (e.g., CubiCAR), which contains a marker for detection and selection, suicide gene, and CAR in a single construct [87, 88].

#### Truncated human epidermal growth factor receptor (EGFRt)/Cetuximab

The strategy utilizing EGFRt was developed to ablate CAR-expressing cells as an alternative to CD20-based safety switches [54, 55]. Like CD20, EGFRt is a membrane protein recognized by EGFR antibodies (e.g., FDA-approved monoclonal antibody Cetuximab), making the transduced cells highly amenable to elimination by ADCC. In another approach, a cryptic EGFR epitope 806 was incorporated into the human folate receptor 1 (FOLR1), allowing the elimination of transduced CAR T cells with CH12 monoclonal antibody–drug conjugates (Fig. 2C) [59].

#### Cytosine deaminase (CD)

The *E. coli* or fungal-derived CD catalyzes the hydrolytic deamination of cytosine to uracil. CD can also convert the commonly used antifungal drug 5-fluorocytosine (5-FC) into chemotherapy drug 5-fluorouracil (5-FU). Since the subsequent cellular metabolites of 5-FU inhibit nucleic acid synthesis in proliferating cells and lead to cell death, ectopically expressed CD can serve as a potent suicide gene approach (Fig. 2A). However, compared to HSV-TK/GCV system, the cell killing is much slower due to the slower cellular uptake of 5-FC. In vitro studies also demonstrated that 5-FU metabolites do not require cell–cell junction for a bystander effect [89–92]. CD was clinically tested for cancer treatment; nevertheless, it has not yet been reported as a suicide switch in CAR T cell therapies. Moreover, as a xenoantigen, CD is also likely immunogenic and thus incompatible with cell-based therapies that require long-term persistence [56].

#### Death signalobody, “SFas”

The high affinity chimeric antibody-based receptor death switch (death signalobody) represents another promising concept in suicide genes. So called SFas consists of the extracellular anti-fluorescein scFv domain, the D2 and modified transmembrane domains of the erythropoietin receptor, and the intracellular apoptotic signal-transducing domain of Fas. The apoptosis of cells modified with SFas is triggered by low concentrations of fluorescein labeled BSA and results in the nearly complete elimination of cells in vitro [57]. The system can be potentially engineered with another antibody fragment, which would provide more freedom to choose suitable antigen–antibody combinations, and thus, greater versatility for desired cell therapy applications.

#### Uridine metabolic switch

Most current safety strategies rely on stable ectopic expression of transgenes. As a novel concept, CRISPR/Cas9 gene-editing technology provided an elegant way of controlling cell growth by disrupting uridine monophosphate synthetase (UMPS), the key cellular enzyme in the pyrimidine synthesis pathway. Without UMPS, engineered cell proliferation becomes entirely dependent on the external uridine supply. In addition, the same genetic modification confers resistance to 5-fluoroorotic acid, which can be used to select UMPS knockout cells. This promising approach was successfully tested in various cell lines, pluripotent stem cells, and primary human T cells [58].

## Regulation of CAR gene expression

**Fig. 3:**
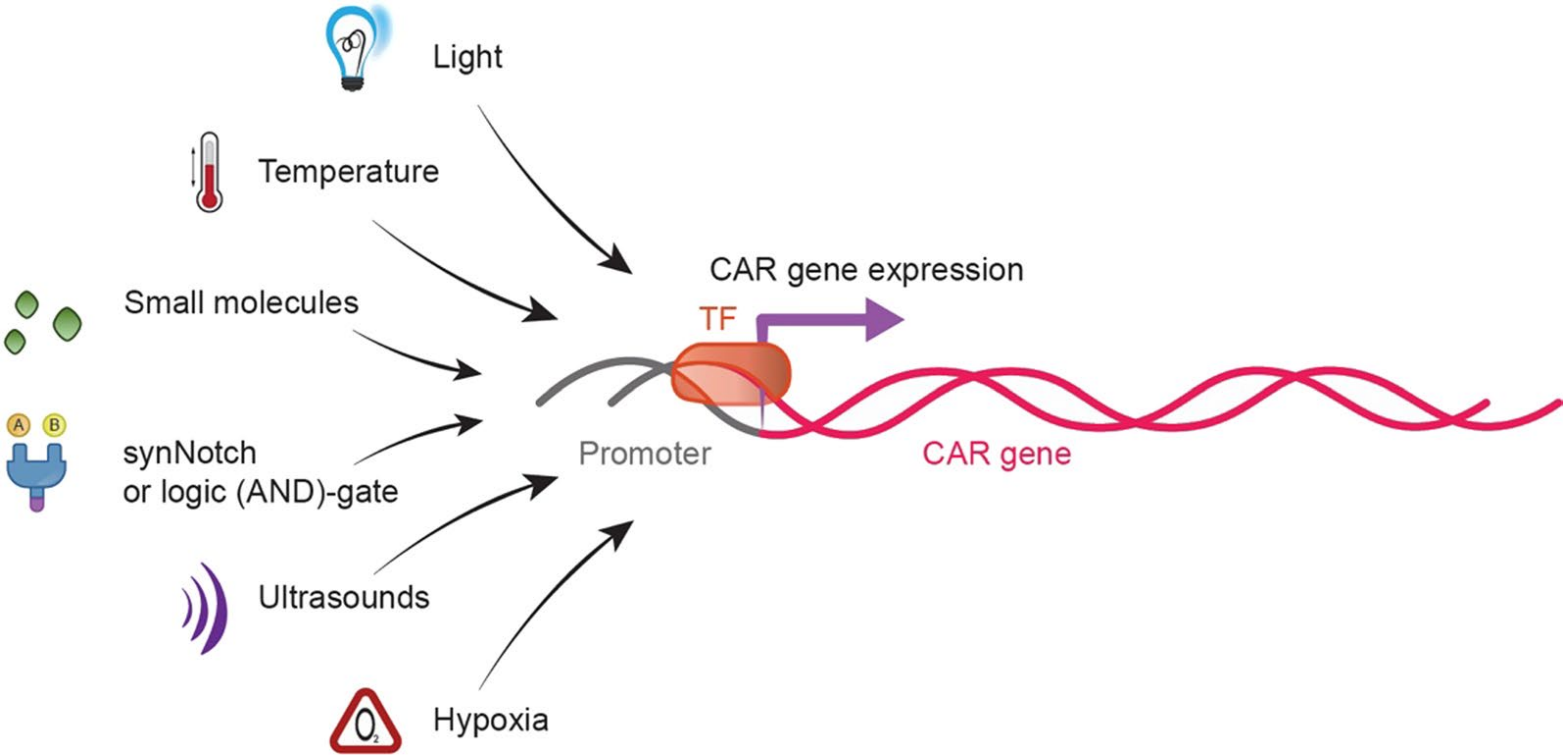
An overview of strategies used for inducible control and modulation of CAR mRNA expression. TF, transcription factor

Eliminating CAR T cells by suicide genes and safety switches constitutes a remedy to excessive adverse reactions but results in permanently losing therapeutic cells. In the following sections, many effective alternative strategies to regulate the proliferation and activity of therapeutic cells at CAR expression and function levels effective alternative strategies will be explored. One of the most popular sets of strategies aims to control the total number of CAR molecules present on the T cell surface via modulating the expression of CAR mRNA by chemical or biophysical inducers (Fig. 3).

### Small molecule mediated regulation of CAR expression

Several clinically approved small chemical molecules can be successfully repurposed for use in artificial gene expression systems to switch the expression of transgenes ON or OFF.

#### Tetracycline (Tet) inducible system (Tet-ON)

Tet-ON is a widely used technology that allows precise, reversible, and efficient control of gene expression with the antibiotic Tet or doxycycline (Dox). Briefly, in the presence of the drug, the engineered reverse tetracycline-controlled transactivator (rtTA) transcription factor binds to the Tet-responsive element (TRE) promoter and initiates gene transcription in a dose-dependent manner. Several CAR T cell platforms, including CD19 CAR [93, 94] and CD38 CAR [95], successfully incorporated the Tet-ON system and demonstrated dose-dependent Dox-induced CAR expression and anti-tumor activity in vitro and in vivo. Like many other gene-inducible systems, the Tet-ON system might also suffer from background “leaky” expression in the absence of an inducer, potentially limiting widespread use for clinical applications. However, researchers continuously work on further improvement of the Tet-ON system’s tightness and fold-response to make it more suitable for CAR T cell therapy [96].

#### Resveratrol (Res) controlled CAR expression

The natural compound Res present in red wine, grapes, and berries has gained attention and popularity as a safe dietary supplement and potential drug candidate [97]. Like Tet in the Tet-ON system, binding of Res to Res-responsive transcriptional transactivator or transrepressor can switch the transgene expression ON or OFF in a dose-dependent manner. The feasibility of this approach to control the expression of CD19 CAR in T cells in vitro and in vivo was successfully demonstrated with intraperitoneal and oral Res administration [98].

#### Zinc finger transcription factor-based inducible switch system

Another promising strategy for small molecule-inducible control over effector functions of CAR T cells is focused on zinc finger transcription factor. In this system, the administration of drugs endoxifen or tamoxifen to engineered T cells activates the artificial zinc finger-based transcription factor to induce gene expression. In vitro and in vivo experiments demonstrated precise induction of CD20 CAR expression with tamoxifen in primary T cells, which was accompanied by rapid induction of CAR T cell effector functions [99].

### Spatiotemporal control of the CAR gene expression

Spatiotemporal confinement and activation of therapeutic cells in the body represent one of the critical outstanding challenges in cell immunotherapy. While small molecule-based inducible expression strategies offer simple, fast, and flexible control, it is difficult to achieve tissue specificity and temporal resolution due to cell permeation and diffusion kinetics. Therefore, an efficient spatiotemporal control approach enabling precise and on-point action of CAR T cells might be groundbreaking, especially for developing improved solid tumor cell therapies. Several strategies based on synthetic biology and biophysical methods were invented to control CAR expression in a specific tissue, thus addressing “on-target, off-tumor” toxicity often associated with CAR T cell products.

#### Light-inducible nuclear translocation and dimerization (LINTAD) system

Various optogenetic protein dimerizer approaches rely on plant-derived light-sensing domains of photoreceptors mediating light-dependent protein–protein interactions – e.g., the light-inducible nuclear localization signal (NLS) technology (LINuS) based on the LOV2 domain of *Avena sativa* phototropin 1 [100] or the CRY2/CIB dimerizer platform derived from *Arabidopsis thaliana* cryptochrome 2 (*At*CRY2) and its light-dependent interaction partner CIB1 [101]. The LINuS and CRY2/CIB technologies were further developed and combined to create the rapid, reversible, and highly sensitive LINTAD ON-switch system, which provides an elegant solution to controlling CAR gene expression and thus confining the effector functions of CAR-expressing T cells within the tissue space illuminated by blue light [102]. The LINTAD system comprises three components: In the dark state, the LexA-CIB1-biLINuS (LCB) protein stays in the cytoplasm, while the CRY2-VPR (CV) transcription activator protein stays in the nucleus. Upon blue light stimulation, the LCB complex unfolds, exposing its NLS motif leading to LCB nuclear translocation and formation of LCB-CV dimer. The LCB-CV complex binds to the LexA BS cassette in the CAR gene promoter, triggering the target CAR gene transcription. As a proof of concept, the LINTAD system was successfully tested in vitro and in vivo using CD19 CAR T cells and leukemic B cell-derived xenografts (CDX) [102]. However, limited light penetration into deep tissues remains a significant challenge.

#### A remote-controlled mechanogenetics (ReCoM) system

ReCoM is another emerging modular method to trigger tissue-specific expression of CARs remotely and noninvasively in T cells by using a combination of ultrasound and mechanosensory proteins [103]. The ultrasound-sensitive Piezo1 ion channel (mechanosensor) naturally expressed by T cells was integrated with ectopically expressed engineered genetic circuits (genetic transducer) to translate the mechanical signal into transcriptional gene activation. Furthermore, when combined with microbubbles adhered to the cell surface that amplify the low-frequency ultrasound waves, Piezo 1 can be activated in tissues at several centimeters. The resulting calcium entry through opened Piezo1 ion channels triggers the downstream cellular signaling pathways, including calcium-sensitive phosphatase calcineurin, which dephosphorylates and activates the transcription factor NFAT. The nuclear-translocated NFAT binds to upstream response elements to initiate CAR gene expression through one-stage or two-stage genetic transducing modules. Primary T cells modified with the ReCoM technology could remotely sense the ultrasound wave and transduce it into transcriptional activation for the CAR expression to recognize and eradicate target tumor cells [103]. Despite the successful pilot study, the requirement for microbubbles as ultrasound signal amplifying cofactors seems to limit the ReCoM method application in vivo.

#### Thermal switch CARs

At least two distinct approaches employing thermal control of CAR expression have been reported. One example of a synthetic thermal-specific gene switch approach is utilizing thermal energy from near-infrared light converted by plasmonic gold nanorods to induce expression of the CAR gene [104]. In this **photothermal switch CAR** approach, T cells are engineered to trigger the expression of CAR and other genes (such as IL-15) in response to mild (40–42 °C) temperature elevations. The expression cassette under the control of the heat-shock protein (Hsp) promoter can dynamically upregulate gene expression more than 20-fold at 42 °C in primary T cells in vivo [104]. However, the main limitation of this approach is the need for the exogenous plasmonic transducer to convert light to heat.

Local heat generated by focused ultrasound (FUS) represents an elegant acoustogenetics method that allows inducible and reversible CAR expression in T cells at the desired time and location without requiring additional exogenous cofactors [105]. Briefly, the thermal energy generated by short pulses of magnetic resonance imaging-guided FUS is coupled with the activation of the CAR expression cassette under the control of the Hsp promoter. The **FUS-CAR system** comprises a Cre-lox gene switch that utilizes Hsp-driven Cre recombinase to activate CD19 CAR gene expression. When expressed, the Cre recombinase removes the lox-flanked ZsGreen-STOP sequence, resulting in sustained production of the CD19 CAR with ZsGreen expression switched on [105]. The photothermal switch CAR and FUS-CAR technologies show the most promising applications in suppressing solid tumor growth in vivo with a substantially improved safety profile over conventional CAR T cells. Notably, thermally controlled promoters can also be used for local expression of other proteins, such as cytokines, to improve the function of therapeutic cells further.

#### Hypoxia sensing CAR switches

The solid tumor microenvironment is often characterized by inadequate oxygen supply. Thus, intratumor hypoxia can be exploited to control the expression of CAR, specifically in the tumor tissue, to effectively mitigate on-target/off-tumor toxicities often associated with CAR T cell therapies [106, 107]. For example, **hypoxia-induced CAR (HiCAR)** combines two oxygen-sensing mechanisms. First, the anti-CD19, anti-AXL, or anti-HER2 CAR expression cassettes are driven by hypoxia response elements upstream of EF1α promoter (chimeric HRE-EF1α promoter, chHE) exhibiting dismal basal expression under normoxia but strong expression under hypoxia. Second, CAR was fused to an oxygen-dependent degradation domain that is actively degraded under normoxia but stabilized under hypoxia. The resulting chHE-HiCAR T cells demonstrated significantly increased cytotoxicity against tumors under hypoxia compared to normoxia in vitro and anti-tumor efficacy comparable to conventional CAR T cells in vivo [108]. A very similar dual-oxygen sensing approach was also used to develop **HypoxiCAR T cells** with inducible expression of pan-anti-ErbB (T4) CAR, which also co-expressed a chimeric cytokine receptor (4αβ) [109]. HypoxiCAR T cells demonstrated high anti-tumor efficacy without off-tumor toxicity in HN3 and SKOV3 CDX mouse tumor models.

#### Synthetic Notch (SynNotch) logic (AND)-gate CAR technology

SynNotch CAR system illustrates a rather distinct sophisticated approach to spatiotemporal control of CAR expression and reducing on-target/off-tumor toxicities of T cell therapies via better separation of tumor and normal cells. Instead of being triggered by biophysical methods, the SynNotch CAR system is an artificial dual-receptor genetic circuit that requires a combination of two different antigens (AND-gate) to initiate CAR expression and activate T cell cytotoxicity. The first step of the AND-gate signaling circuit requires activating an engineered synNotch receptor recognizing the first antigen A. Antigen A recognition leads to cleavage of synNotch and release of an orthogonal transcription factor that activates the expression of CAR directing T cells toward a second tumor antigen B [110–113]. The original system was further modified to accommodate the recognition of various tumor antigen combinations such as ROR1/EpCAM or ROR1/B7-H3 in the breast cancer model [114], GD2/B7-H3 in neuroblastoma [115], EGFRvIII SynNotch that induces expression of EohA2/Il13Ra2 tandem CAR for treatment of glioblastoma [116], or ALPPL2 combined with MCAM or HER2 synNotch CAR combinatorial antigen circuits [117]. Moreover, synNotch-regulated CAR expression averts tonic signaling and T cell exhaustion in vivo, further improving CAR T cell efficacy [116].

### Inducible CAR protein degradation

**Fig. 4:**
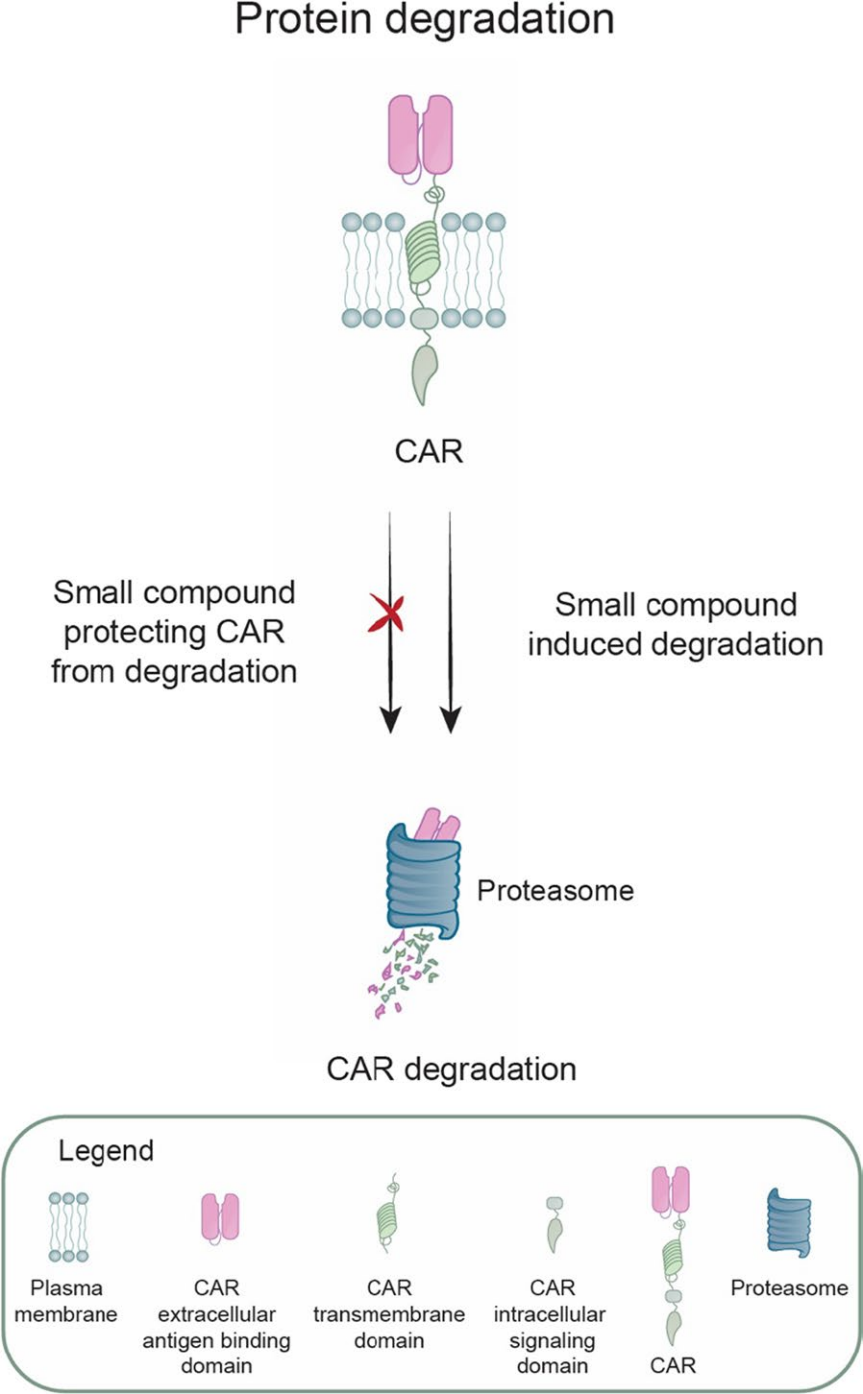
A schematic overview of strategies developed for inducible CAR receptor degradation. CARs with incorporated degradation domains might be degraded or protected against degradation by a small molecule drug. In other technologies, a CAR-inhibiting protein is co-expressed with CAR to regulate CAR function based on the presence or absence of a small molecule

Since only a fully assembled cell surface CAR receptor can activate cytotoxic T cell functions, other rational strategies for controlling CAR T cell activities implement mechanisms for modulating CAR surface presentation, turnover, and stability. In particular, the approaches for rapid and reversible control of CAR protein degradation provide much-desired flexibility to manage therapy-associated toxicities in the case of T cell hyperactivation without CAR T cell removal. These strategies incorporate various small molecule inducible CAR receptor degradation systems to stop CAR-mediated signal transduction (Fig. 4).

#### ON/OFF switches based on Asunaprevir (ASV) and hepatitis C virus N53 protease (HCV-NS3)

As a proof of concept, reversible CAR degradation strategy, the switch-off (SWIFF) CAR technology integrates the target site of HCV-NS3, HCV-NS3, and a degradation moiety (degron) into the intracellular part of the CAR construct (SWIFF-CAR) [118]. Under steady conditions, the HCV-NS3 protease and degron are cleaved and degraded in proteasomes. As a result, the CAR protein is expressed and delivered to the cell surface (ON state). However, blocking HCV-NS3 protease with the specific clinically approved inhibitor ASV prevents cleavage of the degradation domain from the CAR, and therefore the entire CAR protein is targeted for proteolysis (OFF state) [118]. In a similar approach, Cao and colleagues [119] devised the ASV ON-switch CAR technology in which the HCV-NS3 protease domain is embedded between the scFv and the hinge domains of the CAR. This results in constitutive cleavage and deactivation of CAR. However, in vitro or in vivo administration of ASV blocks cleavage and stabilizes CAR; therefore, ASV ON-switch CAR T cells can be repeatedly switched on and off in a dose-dependent manner to effectively eliminate tumor cells as needed [119].

#### ON/OFF switches based on proteasomal degradation induced by lenalidomide

A slightly different chemical genetic technology achieves CAR degradation by incorporating IKZF3 zinc finger degron into the CAR construct [120, 121]. Upon treatment with the clinically approved drug lenalidomide (or other thalidomide analogs), the degron motif interacts with CRBN E3 ubiquitin ligase, which ubiquitinates the fusion protein and targets it for proteasomal degradation [122]. This system was readily used in vivo as an efficient and reversible OFF-switch CAR and further improved by a newly identified super-degron (ZFP91-IKZF3), which is 100-fold more sensitive to lenalidomide [121].

#### Inducible CAR degradation with the proteolysis-targeting chimaera (PROTAC)

Finally, yet another strategy for inducible CAR degradation exploits the PROTAC technology. Lee and colleagues modified the anti-CD19-CAR construct with BRD4-derived bromodomains, targeted by PROTAC compounds ARV-771 and ARV-825 to induce reversible protein degradation by VHL and CRBN E3 ubiquitin ligases and a proteasome pathway [123]. Interestingly, the BRD4 inhibitors also suppress leukemic growth. Thus, a single dose of PROTAC compounds regulates CAR T cell functions and, at the same time, inhibits tumor growth [123].

## Inducible formation of a functional CAR or inducible CAR disassembly

**Fig. 5:**
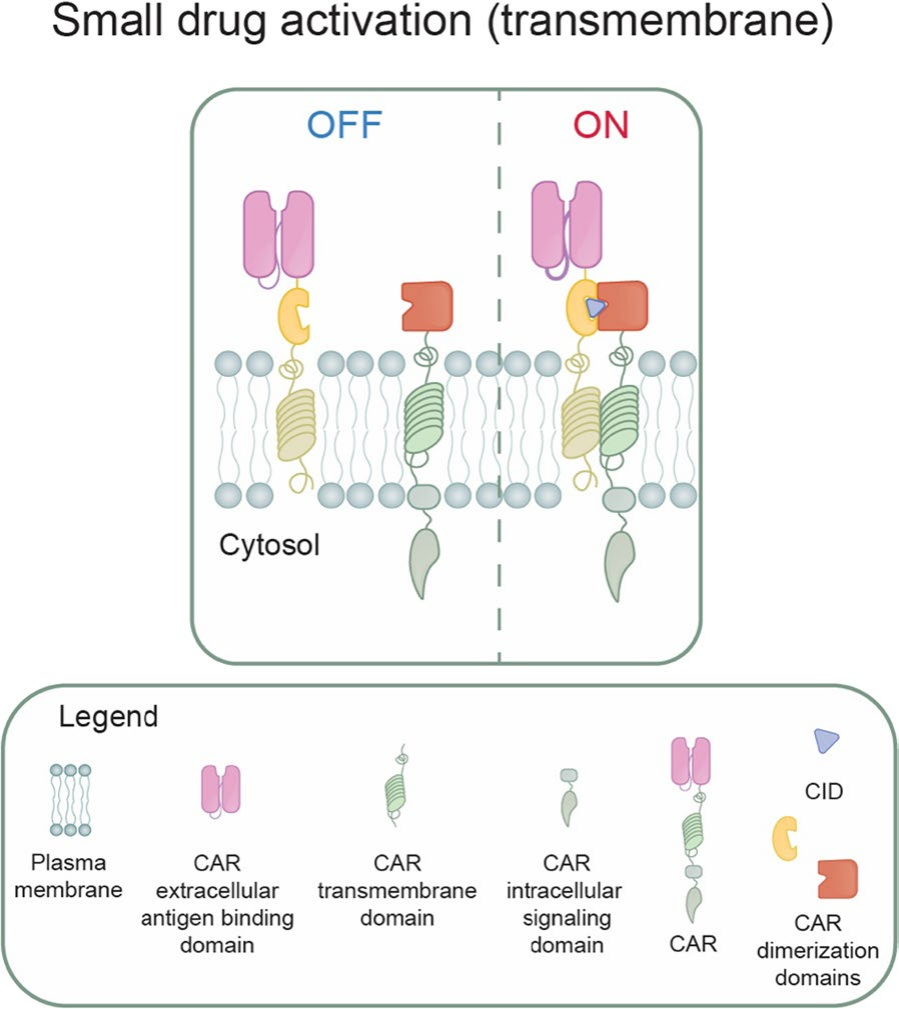
Inducible formation of a functional CAR or inducible CAR disassembly. The CAR is split into two parts that associate (ON-switches) or dissociate (OFF-switches) upon activation by small molecule drugs. CID, chemical inducer of dimerization

Among many strategies developed to control the function of CAR T cells, the inducible assembly or disassembly of a functional CAR represents one of the earliest approaches (Fig. 5). The CAR receptor split into two constitutively expressed proteins is modified with dimerization domains that can interact and form a functional CAR only in the presence of dimerizing drugs (ON-switches). Alternatively, the two parts of CAR may associate constitutively and be disrupted on demand by a small molecule (OFF-switches). The concept of splitting critical components from the CAR was inspired by the natural process of T cell activation, which requires the co-engagement of TCR with peptide:MHC complex (“first signal”) and concomitant activation of costimulatory receptors (e.g., CD28 or 4-1BB, “second signal”). In a conventional CAR construct, the TCR and costimulatory signaling modules are artificially present in a single polypeptide. However, thanks to the split CAR design separated into two distinct polypeptides containing heterodimerization domains, the antigen-dependent T cell activation occurs only in the presence of a dimerizing drug [124–126]. Various in vitro and in vivo studies using split CAR designs with intracellular [124] or extracellular [125] heterodimerizing domains demonstrated the feasibility and high efficiency of these technologies. While the original CAR switches were suboptimal for therapeutic applications due to the non-human origin of protein components [124], more recent approaches rely on safer human-derived protein dimerization domains and clinically approved drugs as dimerization agents [126]. This chapter will explore various approaches and discuss their utility in CAR T cell immunotherapies.

The inducible formation of a functional CAR receptor was originally described as **ON-switch split CAR** [124]. The authors found an ideal way of splitting and modifying a conventional anti-CD19 CAR construct into two parts retaining a high signaling capacity upon antigen binding and domain heterodimerization. The first part of the split CAR contains the extracellular antigen binding domain (scFv), CD8 hinge and transmembrane domain, 4-1BB costimulatory motif, and a heterodimerization domain. The second part consists of the DAP10 ectodomain and CD8 transmembrane domain, 4-1BB costimulatory motif, CD3-zeta signaling chain, and a heterodimerization domain. This ON-switch CAR design worked equally well in vitro and in vivo with two different dimerization modules: (A) FKBP-FRB* module heterodimerized by rapamycin analog AP21967, and (B) the gibberellic acid heterodimerization module (using Arabidopsis GID1 and GAI domains) [124].

A similar split CAR approach denoted as the **dimerizing agent–regulated immunoreceptor complex (DARRIC)** also consists of two physically separated membrane-bound components equipped with FKBP12-FRB* dimerization module, but in this case, in their extracellular parts [125]. One polypeptide consisted of antigen targeting scFv (eg., CD19, BCMA), FKBP12, and CD4 transmembrane domain. The other polypeptide contained FRB*, CD8 transmembrane domain, and intracellular signaling domains of 4-1BB and CD3 zeta. The addition of rapamycin or rapamycin analog AP21967 led to rapid DARRIC T cell expansion and killing of target tumor cells in cocultures in vitro. DARRIC T cells exhibited superior in vivo activity compared to normal CAR T cells, even at low non-immunosuppressive rapamycin concentrations. Interestingly, the DARRIC system can be modified to expand antigen recognition diversity of CAR T cells either by using soluble rapamycin-prebound scFv-FKBP12 polypeptide (DARRIC plug-in) or by inserting FRB* into a standard CAR (adaptable CAR T) leading to the retargeting of the original CAR to administered DARRIC plug-in CAR [125].

Furthermore, the **Avidity-controlled CAR (AvidCAR)** platform exploits the natural avidity effects of antigen recognition to develop CAR constructs with a drug inducible ON-switch function [127]. In this proof-of-principle approach, the low-affinity CAR is based on mutants of the hEGFR-binding protein rcSso7d or hHER2-specific binder zHER2-AK, whose functions strictly depend on bivalent antigen interactions. In addition, the low-affinity CAR-incorporated FKBP12-FRB heterodimerization module allows regulation of CAR T cell function by the small molecule AP21967. The AvidCAR system was also adapted for AND-logic control function to recognize two different surface antigens or surface and soluble antigens, thus improving tumor specificity and solid tumor targeting [127].

The continuous research into CAR ON-switches ultimately has led to the developing of alternative, highly efficient systems engineered from nonimmunogenic human proteins controlled by non-immunosuppressive FDA-approved drugs. One example of such a novel design is the **lenalidomide-gated ON switch split CAR** [121]. The system originates from the OFF switch based on proteasomal degradation induced by lenalidomide, as discussed above. In this case, however, lenalidomide and other thalidomide analogs mediate strong interaction between engineered CRBN mutant and IKZF3 zinc finger domains instead of inducing protein degradation. The switch CAR is derived from a conventional CD19 CAR split into two transmembrane polypeptides with intracellular dimerization domains. The resulting ON-switch split CAR T cells were effectively controlled in vivo by subtherapeutic lenalidomide doses to induce rapid and reversible antitumor activity, albeit at a slightly lower potency than conventional CAR T cells [121].

Similarly, the **lipocalin-based conformation-specific ON-switch CAR** system [126] was designed from human lipocalin retinol-binding protein 4 (hRBP4), which undergoes a conformational change upon the binding of the small hydrophobic orally available drug A1120. The A1120-induced structural change of hRBP4 enables interaction with engineered binder scaffolds such as reduced charge Sso7d or the tenth type III domain of human fibronectin. The resulting conformation-specific ON-switch was incorporated into a two-chain CAR system with transmembrane and soluble parts to potently control the activity of primary human CAR T cells in vitro.

As an alternative to split ON-switch CAR strategies, the **FKBP/FRB multichain CAR (FKBP/FRB-mcCAR)** technology was developed by modifying the hinge domain of FcεRI-based CAR scaffold with FKBP and FRB (or FRB*) domains [128]. Interestingly, the integration of FKBP/FRB domains in the mcCAR hinge domain interfered with the surface expression of mcCAR, which could be detected only upon the addition of a dimerizing drug. Therefore, this novel CAR architecture allows turning mcCAR from an OFF-state to an ON-state by controlling the scFv presentation at the cell surface upon the addition of the small molecule rapamycin or non-immunosuppressive rapalog AP21967. As proof of principle, the anti-CD19-FKBP/FRB-mcCAR system was tested in vitro and demonstrated specific target cell killing comparable to constitutively expressed anti-CD19 mcCAR [128].

Interestingly, instead of using small molecule drugs, ON-switch CARs can also be engineered using optogenetic protein dimerizer systems to respond to light stimulation, as demonstrated in the **light-switchable CAR (LiCAR)** design [129]. Briefly, the conventional anti-CD19 CAR was split into the membrane and intracellular parts, each equipped with a photo-responsive plant-derived dimerizer element such as Arabidopsis cryptochrome 2 (CRY2) and the N-terminal region of its photo-sensitive binding partner CIB1 (CRY2/CIBN) or Avena light-oxygen-voltage domain 2 (LOV2) and its binding partner sspB (LOV2-ssrA/sspB). After co-expression of modified CAR subunits, the dimerization and assembly of a functional CAR are achieved with blue light stimulation. Since blue light has minimal tissue penetration, the LiCAR system is complemented by luminescent upconversion nanoparticles, which capture deep tissue penetrating near-infrared light and emit LiCAR-activating blue light. Extensive in vitro and in vivo testing of LOV2-based LiCAR in diverse mouse models demonstrated highly efficient and tunable spatiotemporal control over CAR T cells. Compared to conventional CARs, the LiCAR system shows substantially improved safety profiles (“on-target, off-tumor” cytotoxicity and cytokine release syndrome), which makes LiCAR an attractive therapeutic modality for further development and clinical evaluation [129].

A slightly different concept to engineering an ON-switch CAR was applied in the **Chemically Regulated SH2-delivered Inhibitory Tail (CRASH-IT) switch**, which exploits natural signaling modalities of immunoreceptors [130]. Rather than degrading or disrupting the CAR receptor, the CRASH-IT switch recruits negative regulators of immunoreceptor signaling to CAR in a drug-controlled manner. The CRASH-IT switch is an engineered intracellular adaptor protein consisting of SH2 domains of Zap-70 binding ITAMs and inhibitory domains of PD-1 for the recruitment of protein phosphatases inhibiting immunoreceptor signaling. Moreover, the Zap70-PD1 scaffold was fused with a Small Molecule-Assisted Shutoff (SMASh) tag to achieve tight pharmacological control. In the basal state, HCV protease cuts off the SMASh tag linker; thus, Zap70-PD1 can block the CAR activation. However, adding the HCV protease inhibitor ASV prevents SMASh tag cleavage, and the CRASH-IT switch degrades the proteasome [130]. In addition to effectively regulating CAR activation, the CRASH-IT platform can be potentially applied to any cell therapy product involving ITAM-containing receptors (e.g., TCR therapy).

As opposed to ON-switch CAR designs, several OFF-switch genetic systems were developed to modulate CAR T cell activity at the level of CAR disassembly. For instance, one approach, denoted **STOP-CAR**, is based on a rational computer-assisted design of a chemically disruptable heterodimer originating from two human protein scaffolds (Bcl-XL and the BH3 domain of Bim) [131]. The CAR is split into two self-assembled chains, which can be disrupted on demand by small-molecule high-affinity drugs. The experimentally tested in vitro and in vivo efficacy equal to conventional CARs, the use of human-derived proteins with low immunogenicity, and drugs with long half-life promise to advance this system into clinical development. Similarly, another OFF-switch split-CAR control system, denoted **minocycline TetCAR**, exploits the spontaneous heterodimer formation between tetracycline repressor protein B and a small peptide tetracycline mimic, disruptable with the FDA-approved small molecule antibiotic minocycline [132]. Successful TetCAR testing in vitro and in vivo demonstrated that minocycline regulates TetCAR T cell activity in a dose-dependent and reversible manner.

Finally, a slightly different technology of conditional CAR modulation, called **CondCAR OFF-switch**, implements antibody scaffolds with inducible binding affinities [133]. In CondCAR, the engineered antigen recognition site of single-chain anti-CD33 CAR can be transiently and reversibly attenuated by the FDA-approved drug methotrexate in cases with excessive CAR T cell cytotoxicity, thus substantially improving the safety profile of therapeutic cells. Moreover, CondCAR T cells demonstrated in vitro and in vivo cytotoxic activity comparable to conventional CAR T cells [133].

## Inhibitory CARs

**Fig. 6:**
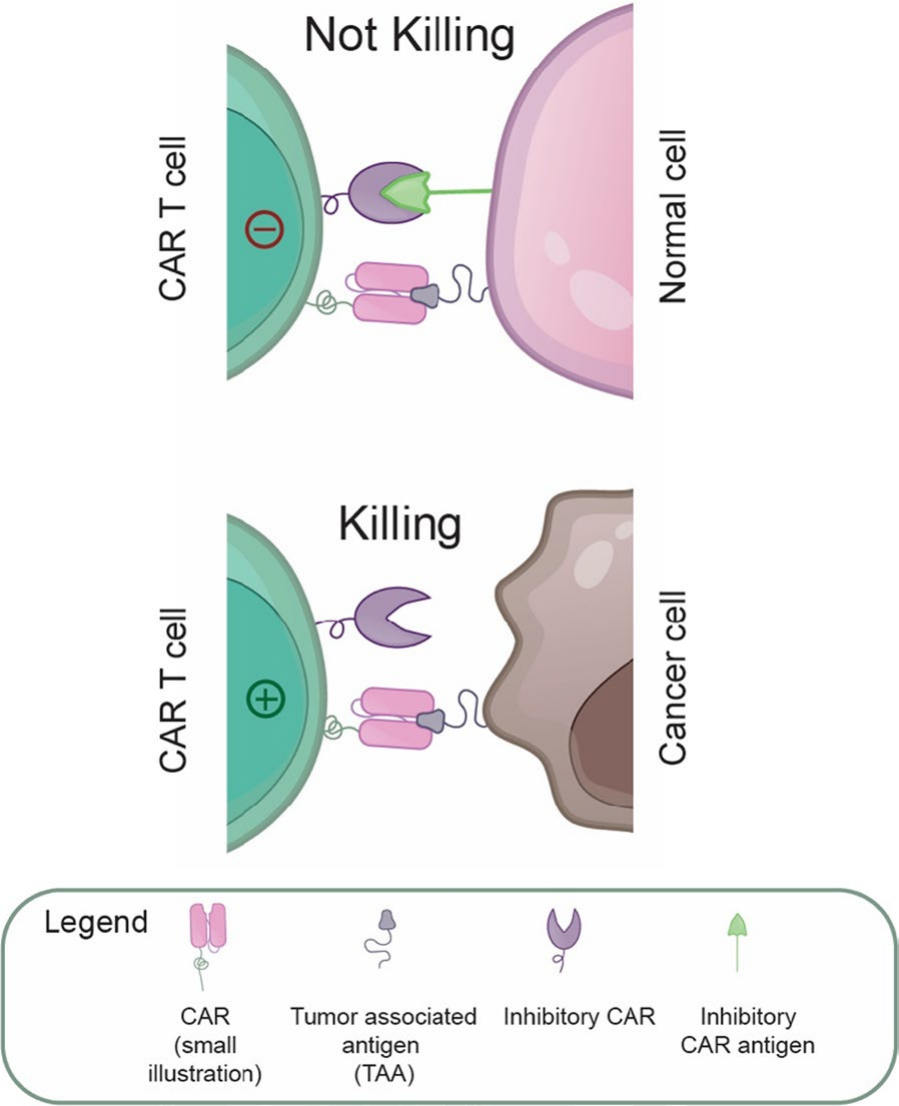
Inhibitory CAR receptors. When co-expressed with conventional CARs, the inhibitory CARs prevent CAR T cells from killing normal cells with target antigen expression, thus increasing the specificity of CAR T cells

One area of intensive CAR development focuses on solving the problem of the “on-target, off-tumor” toxicity of CAR T cells to healthy cells and bystander tissues with target antigen expression [14, 15, 26, 27]. For instance, in the case of CD19-targeted CAR T cell therapies in B cell malignancies, one of the most common side effects is B cell aplasia [14, 26]. B cells express high levels of CD19, and conventional CD19 CAR T cells cannot discriminate between tumor and normal cells. Thus, to overcome this challenge, several so-called inhibitory CARs operating on the principle of NOT-gate Boolean logic were designed. In these combinational targeting approaches, CAR T cells are equipped with the CAR targeting tumor-associated antigens (TAAs) and a second inhibitory CAR specific for a distinct antigen present in normal healthy cells, which blocks the killing program upon antigen recognition (Fig. 6). The inhibitory CARs are composed of an extracellular antigen recognition domain (usually scFv antibody fragment) and intracellular inhibitory signaling domains derived from inhibitory immunoreceptors such as CTLA-4 [134–136] or PD-1 [134–138]. Mechanistically, the inhibitory domains contain immunoreceptor tyrosine-based inhibitory motifs, which recruit protein phosphatases, the principal negative regulators of immunoreceptor signaling, and thus counteract the T cell activating signals originating from CARs [134–138]. As a result, the inhibitory CAR strategy allows more selective cancer cell recognition and elimination without potential collateral damage to normal healthy cells and tissues. Interestingly, this artificial genetic engineering approach mimics the function of NK cells in processing the signals from activating and inhibitory receptors.

The first proof-of-concept inhibitory CAR design, called **iCAR**, was constructed from scFv targeting the prostate-specific membrane antigen (PSMA) fused to the transmembrane segment and signaling domains of CTLA-4 or PD-1 [134]. When co-expressed with conventional anti-CD19 CAR in primary human T cells, iCAR permitted effective discrimination between target and off-target cells in vitro and in vivo. Moreover, the mere presence of iCARs in T cells did not affect their proliferative and tumor cell killing capacity in the absence of inhibitory antigen, and the system was fully reversible after the engagement of iCARs [134]. Therefore, iCAR protects against inadequate CAR T cell specificity; however, it does not compromise the CAR T cell functionality.

The application potential of iCAR technology was further corroborated by a similar approach to mitigate the on-target off-tumor toxicity of novel CD93-specific CAR T cells in acute myeloid leukemia (AML). The CD93 CAR T cells demonstrated adequate leukemic clearance and minimal hematopoietic toxicity; however, with substantial off-target toxicity to endothelial cells with shared CD93 antigen expression (139). The feasibility of iCAR NOT-gated CAR T cells for sparing endothelial cells was tested using an in vitro model cell line with CD19 expression. The CD19 iCAR containing PD-1 or TIGIT inhibitory domains potently inhibited CD93 CAR T cell activation in the presence of CD19 and CD93 antigens on endothelial cells [139]. These encouraging results warrant the future utility of iCARs, which will go hand in hand with discovering suitable targets for NOT-gated combinatorial immunotherapies.

**Double-Arm CAR** is another inhibitory CAR that works as a NOT-logic gate [140]. Double-arm CAR consists of two distinct proteins, so-called ‘Signal-CAR’ and ‘Scissors-CAR”. Signal-CAR recognizes the tumor antigen on the cancer cell surface, while Scissors-CAR recognizes another antigen on healthy cells. Scissors-CAR contains a protease domain that cleaves Signal-CAR. Therefore, double-arm CAR T cells kill only cells that express the Signal-CAR target antigen but do not express the Scissors-CAR target antigen. Double-arm CAR was proved in a model with anti-CD19-Signal-CAR cleaved by the anti-HER2-Scissors-CAR [140].

In addition to markedly increasing the specificity of conventional CAR-based therapies, inhibitory CARs might allow precise discrimination and targeting of tumor cells with genetic AND/NOT signal integrators. In particular, the clonal loss of heterozygosity (LOH) in a large majority of cancer cells provides a unique therapeutic window that can be exploited by reprogrammed immune cells. One approach took advantage of downregulated expression of human leukocyte antigen HLA-C1 to discriminate between normal cells (HLA-C1 positive) and tumor cells (HLA-C1 negative). HLA-C1 is naturally recognized in normal cells by the inhibitory receptor KIR2DL2, predominantly expressed in NK cells. Thus, an inhibitory CAR, denoted **iKP (KIR2-PD1) CAR**, was engineered using the extracellular domain of KIR2DL2 and the intracellular domain of PD-1 [137]. When co-expressed with CD19 CAR in T cells (iKP-19-CAR T), iKP CAR recognizes HLA-C1 on normal cells and delivers strong negative signaling to inhibit CAR T cell response via the PD-1 inhibitory domain. Meanwhile, in the absence of HLA-C1 on tumor cells, iKP CAR remains inactive and allows tumor cell elimination by CD19 CAR in vitro and in vivo [137]. Interestingly, iKP-19-CAR T cells maintained a more naïve phenotype and a higher proportion of central memory T cells. These results demonstrated the potential of inhibitory CARs to prevent B cell aplasia associated with current CD19-targeted CAR T cell therapies.

Other promising approaches denoted as **T-cell module 2 (Tmod2)** [135] and **Neoplasm-targeting Allele-Sensing CAR (NASCAR)** [136], applied inhibitory CAR technology to exploit LOH in cancer. The Tmod2 system targets CD19 antigen with a conventional CAR and HLA-A*02 (LOH frequency of 13%) with inhibitory CAR composed inhibitory domains of the LIR-1 receptor, which delivered slightly more potent inhibition than respective domains of CTLA-4 or PD-1. Testing Tmod2 in vitro and in vivo proved effective for signal integration and discrimination between normal and tumor cells via HLA locus targeting [135]. Similarly, the NASCAR platform utilizes a combination of CAR and iCAR specific for two common alleles of the HLA-A gene (HLA-A*02:01 or HLA-A*03:01) to distinguish between normal and cancer cells by targeting the HLA-A allele lost through LOH. The high application potential of NASCAR technology was demonstrated in vitro and in vivo [136]. Beyond the utility of inhibitory CARs in T cell-based immunotherapies, this strategy was also employed to enhance the specificity of CAR NK cells reprogrammed to target HLA-DR loss in hematological malignancies. The **anti-HLA-DR iCAR** was co-expressed with anti-CD19 or anti-CD33 activating CARs and mediated highly selective killing of HLA-DR-negative tumor cells in vitro and in a xenograft mouse model [138].

## Modular universal adaptor CAR platforms

**Fig. 7:**
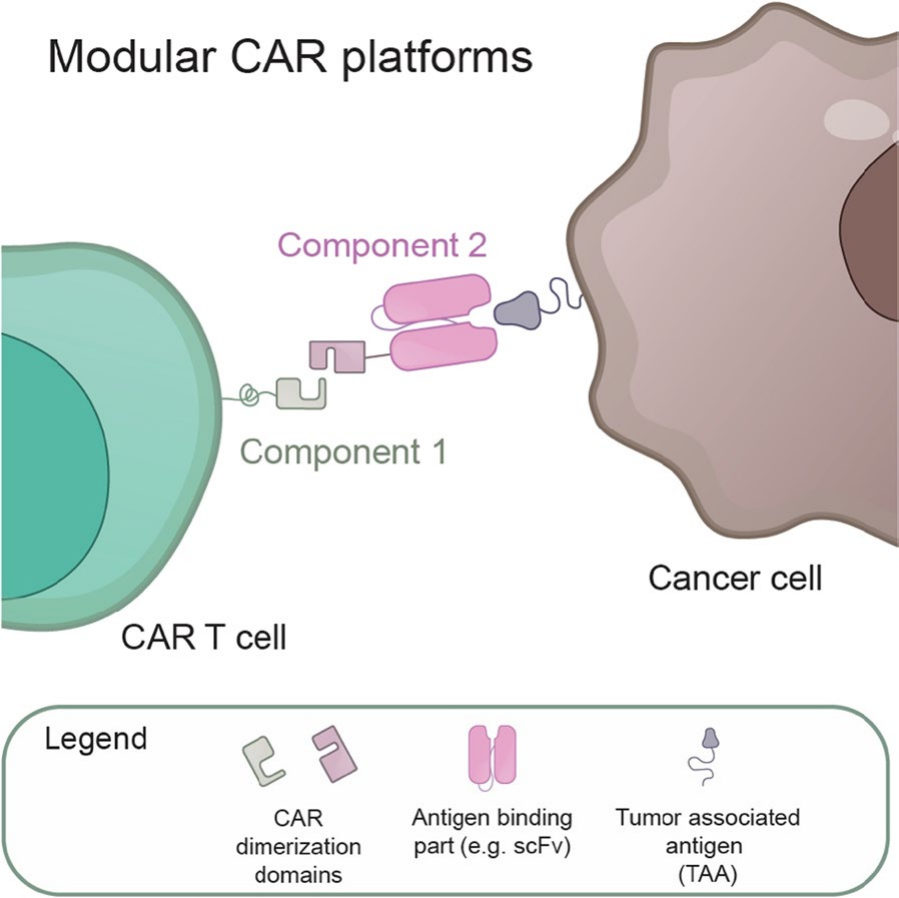
The schematic of two-component adaptor CAR platforms. The soluble adaptor or antigen recognition molecule (component 2) binds to the transmembrane receptor (component 1) at the surface of the engineered T cells to form a functional CAR activating T cell effector functions

The following section will discuss a summary of the expanding body of literature on various other CAR T cell control strategies and their combinations that do not fall into the previous chapters. Most discussed strategies employ modular two-component switchable antigen receptors, often referred to as “adaptor CARs” or “universal immune receptors” (Fig. 7). The first component is usually a constitutively expressed universal transmembrane receptor lacking an antigen-binding domain but equipped with an extracellular module for mediating strong protein–protein or protein–ligand interactions. The intracellular signaling part of the universal receptor is responsible for T cell activation. The second component, the switch, can be a bispecific antibody (bsAb) or other soluble adaptor equipped with antigen recognition domain (e.g., scFv antibody fragment). The adaptor is also usually tagged with a protein interaction domain, peptide, or neo-epitope ligand for noncovalent or covalent interaction with the receptor. Thus, a fully functional antigen receptor is formed only when the second component is administered to engineered T cells expressing the universal receptor. Modular universal antigen receptor platforms represent a practical, uncomplicated way to gain temporal and quantitative control over therapeutic T cells and to precisely regulate their activity, expansion, and cytotoxicity once administered to patients, by simply titrating the antigen recognition component. Moreover, these innovative technologies offer much-desired flexibility in antigen choice and, in the case of antigen escape, T cell retargeting by providing different antigen recognition modules without the need for T cell re-engineering.

### Fc Receptor (FcR)-based antibody-dependent cellular cytotoxicity (ADCC) CARs

The Fc Receptor (FcR)-based CARs belong amongst the first reported two-component CAR platforms. The FcR-based CARs utilize the natural binding of FcγRIIIa (CD16) to Fc parts of IgG antibodies combined with inherent FcR signaling properties to activate T cell cytotoxic functions via ADCC pathway [141]. Moreover, the high flexibility of this approach in target tumor selection is achieved by combining FcR CARs with different therapeutic antibodies. Several FcR-CAR designs were developed and tested in vitro and in vivo [141–144]. For instance, the chimeric FcγRIIIa-FcεRIγ (CD16/γ) or CD16-CD3ζ receptors (cCD16ζ) were successfully used in combination with anti-CD20 mAb rituximab in T cells and NK cells to confer cytotoxicity against malignant B cells [141, 143] or in combination with anti-Her2/neu mAb trastuzumab in NK cell model cell line NK-92 to kill breast cancer cell lines [141].

Similarly, experiments with the second-generation FcR CARs further modified with costimulatory signaling domains of CD28 [144] or 4-1BB [142] proved to be universally functional when combined with various therapeutic antibodies. From a clinical perspective, FcR CARs might be alternative or complementary effectors to enhance the antitumor efficacy of mAb therapies for cancer. However, several clinical trials were terminated or put on hold for commercial and safety reasons.

### Anti-TAG CARs: monoclonal antibody (mAb)-coupled CARs

Many other approaches rely on combining existing therapeutic mAbs with universal transmembrane signaling receptors to create mAb-coupled CARs. Since the antibodies are usually tagged with small molecules or peptides for binding to CAR, this CAR technology is also termed Anti-TAG CAR. Compared to FcR CARs discussed in the previous section, Anti-TAG CARs provide another layer of specificity due to the more restricted interaction of tagged mAbs with the signaling receptor present only in engineered therapeutic cells.

The **Anti-Fluorescein isothiocyanate (Anti-FITC) CAR** is a conventional CAR with specificity for FITC-labelled therapeutic antibodies, which mediate T cell targeting to TAAs [119, 145–148]. The synthetic dye FITC is well-characterized and has been widely used for antibody labeling in flow cytometry applications. Several studies with Anti-FITC CAR T cells demonstrated high proliferation capacity, cytokine production, and cytotoxic activity against various FITC-mAb labeled cancer cells in mouse tumor models, comparable or superior to conventional CARs [145, 148]. The cytotoxic activity of Anti-FITC CAR T cells targeted to Her2 antigen in breast cancer cells was comparable or, when combined with bsAb, even superior to conventional anti-Her2 CARs [119]. Interestingly, Anti-FITC CARs are not limited to combinations with antibodies, as shown in studies using FITC-conjugated folate (folate-FITC, EC17) to target cancer cells overexpressing folate receptor (FR) successfully [72, 146]. Furthermore, the function of anti-FITC CAR T cells can be modulated by titrating the dose of specific FITC-mAbs or deactivated by nonspecific competitive FITC-IgG or sodium fluorescein, thus mitigating the risks of developing severe CRS [72, 145, 147, 148].

Another level of Anti-FITC CAR modulation was recently achieved by including ortho-nitrobenzyl ester photocleavable linker into folate-FITC module [149] or by coupling of FITC-mAbs with photocaging technology, which protects FITC from CAR T cell recognition [150]. These technologies allow the control of Anti-FITC CAR T cell activation and killing with ultraviolet light exposure in a spatiotemporal manner. A potential disadvantage of Anti-FITC CAR technology might be FITC immunogenicity, as the anti-FITC antibodies were detected in sera of mice treated with FITC-conjugated rituximab [145]. Thus, Anti-FITC CAR represents a promising strategy; however, clinical trials are needed to confirm the therapeutic activity and safety.

Other examples of anti-TAG CARs include **Biotin-binding Immunoreceptor (BBIR) CAR** and **Monomeric Streptavidin 2 (mSA2) CAR** platforms based on strong interaction properties of biotin to avidin or its variants [151–153]. In these approaches, which share many similarities with Anti-FITC CAR technology, T cells expressing CARs with extracellular avidin domains are switched on by selective recognition of Biotin-mAb or other biotinylated antigen-binding adaptors. However, the antigenicity of chicken avidin or bacterial streptavidin and the presence of natural anti-biotin antibodies in sera of healthy individuals might potentially hinder the use of such CARs in humans [154]. Another alternative biotin-based strategy, termed **AdCAR system**, utilizes a biotinylated antigen-targeting adaptor and CAR receptor derived from mBio3 scFv, which recognizes biotin in the context of a specific linker. This strategy applied in primary T cells and NK-92 cells was highly effective in eliminating lymphoma cells in vitro and in vivo [155, 156]. Interestingly, the AdCAR system can also be used to detect soluble latent TGF-β and activate T cell effector functions within the tumor microenvironment of a pancreatic tumor model [157].

In addition to chemical modifications with FITC or biotin, antibodies, antibody fragments, or other tumor-targeting receptors can be engineered with non-immunogenic small peptide tags for Anti-TAG CAR systems such as **Peptide neoepitope (PNE) specific CARs** [147, 158–160]. PNE is a short non-immunogenic sequence from yeast transcription factor GCN4 that can be introduced at defined sites of antigen-specific therapeutic antibodies and Fabs to create the switch. The scFv from high-affinity anti-GCN4 then serves as recognition domains of PNE CARs. The PNE CAR technology can be readily adapted with different therapeutic antibodies to target diverse antigens and allows precise control of CAR T cell activity by the dosage of an antibody-based switch [147, 158, 160]. Similarly, the **Convertible CAR (cCAR)** technology leverages the robust binding affinity between the inert form of NKG2D receptor ectodomain to its cognate ligand MICA to engineer a bispecific antigen-targeting adapter molecule (MicA-body^™^), which can also serve as a vehicle for targeted delivery of cytokines in vitro and in vivo [161]. The cCAR technology was successfully tested to kill tumor cells in a mouse lymphoma model [161] and to effectively eliminate HIV-infected cytotoxic T cells and reactivated CD4^+^ reservoir T cells in vitro and ex vivo [162].

Interestingly, a proof-of-concept study adapted PNE CAR technology to enable the targeting of the pocketome fraction of cancer cell surface receptors as **Chemically programmed antibody fragment (Cp-Fab) CAR** [163]. In this approach, the cp-Fab based on the catalytic antibody h38C2 is genetically modified with the PNE motif to bind to PNE CARs, and then chemically programmed with a β-lactam-biotin-folate compound for detection and efficient targeting of the folate receptor 1 (FOLR1), which is often overexpressed in aggressive solid malignancies [163].

Finally, two technologies were employed to link therapeutic mAbs to universal CAR receptors covalently, and both represent up-and-coming innovations of universal Anti-TAG CARs [164–167]. The **SpyCatcher CAR** is based on the SpyCatcher-SpyTag chemistry, which allows a rapid and spontaneous post-translational covalent attachment of targeting ligands to universal CARs. The SpyCatcher CAR T cells were successfully tested in vivo in combination with Herceptin-SpyTag targeting Her2-positive ovarian cancer cells SKOV3 [164] and anti-glypican-3-SpyTag targeting hepatocellular carcinoma cells HepG2 [165]. The other technology, **SNAP-CAR**, achieves covalent bonding of CAR with mAbs using the SNAPtag self-labeling enzyme present in the extracellular part of CAR, which reacts with benzylguanine (BG)-conjugated mAbs [166, 167]. The studies demonstrated SNAP-CAR function in vitro and in vivo with several clinically relevant BG-conjugated antibodies in an antigen-specific and antibody dose-dependent manner. The permanent on-demand programming of universal therapeutic cells with antigen-targeting ligands is a powerful concept overcoming suboptimal CAR functions due to the lower affinity of soluble adaptors to universal CAR receptors. However, the serum half-life of soluble adaptors seems to be short; thus, repeated administration of the switch molecules is still required for a prolonged therapeutic effect [165, 166]. Moreover, since the SpyTag/SpyCatcher system originates from bacterial components, immunogenicity could be another potential limitation [164, 165]. The SNAPtag, on the other hand, was engineered from human O-6-methylguanine-DNA methyltransferase (MGMT), which theoretically reduces the probability of inducing immune reactions [166].

### Universal CARs

In this final section, a summary of the development of CAR technologies, most often referred to as “universal”, including UniCARs, RevCARs, and SUPRA CARs will be discussed. However, these universal CARs can be considered part of the Anti-TAG CAR category as they share similar overall modular CAR logic.

**UniCAR** represents the first reported and most extensively tested universal CAR technology [1, 21, 168]. It originates from the modular bispecific T cell engager (BiTE) UniMAB [168, 169]. The soluble antigen-targeting part of the UniCAR system is equipped with a small peptide epitope tag derived from a non-immunogenic nuclear La/SS-B autoantigen [21, 169–172]. The receptor part of UniCAR is constructed of the extracellular anti-peptide epitope scFv and signaling domains in the intracellular part [168]. Over the years, the original UniCAR system was modified with different tumor antigen-targeting modules to provide an effective therapeutic option for various tumor antigens. Moreover, an improved targeting module crosslinking UniCAR with the costimulatory receptor 4–1BB further enhanced UniCAR T cell expansion, persistence, and effector functions [173]. The UniCAR technology and all its variants demonstrated great therapeutic potential in vitro and in vivo against hematological malignancies and solid tumors, including AML cells expressing CD33 and CD123 antigens [1, 21, 173–175], CD19^+^ tumor cells [176], carcinoma cells expressing tumor-associated carbohydrate sialyl-Tn antigen carcinomas [177, 178], EGFR-expressing tumor cells [179, 180], CD98 or EGFR radioresistant head and neck squamous cell carcinoma cells [181], and prostate cancer cells overexpressing prostate stem cell antigen (PSCA) or PSMA [21, 182, 183]. In addition to application in T cells, the UniCAR technology was proven to work in the NK-92 cell line for the specific killing of neuroblastoma and other disialoganglioside GD2-expressing cancer cells [184].

A similar switchable, split, and adaptable universal CAR platform, termed **RevCAR**, was established using a fully humanized bispecific targeting module (RevTM) and an overall smaller size than UniCARs [185]. Like other universal CAR approaches, RevCAR offers excellent versatility and advanced control options such as combinatorial antigen targeting (AND- and OR- gate). RevCAR-modified and RevTM-redirected T cells were highly effective in eliminating various cancer cells expressing different TAAs in vitro and using mouse tumor models [186], including patient-derived AML cells expressing CD33 and CD123 [187] and glioblastoma cells [188].

**Split, universal, and programmable (SUPRA) CAR** technology also integrates several improved comprehensive features to enhance the specificity, safety, and cell-specific programmability of CARs [189]. The SUPRA CAR system consists of a universal transmembrane receptor fused to a transcription factor-derived leucine zipper adaptor (zipCAR) and a separate soluble antigen-targeting scFv with another leucine zipper adaptor (zipFv) molecule. Upon dimerization of the leucine zippers, the antigen signal is transmitted to the CAR intracellular signaling domain, and the engineered T cells become activated. The power of SUPRA CAR technology was demonstrated in vitro and in vivo using mouse models of leukemia and solid tumors [189]. Later, the SUPRA CAR system was also upgraded to incorporate a co-inhibitory zipCAR with a NOT-logic function and even an advanced three-input logic gate combining AND- and NOT-logic gates. Notably, the versatility and complexity of logically controlled SUPRA CARs was shown by zipFv-mediated programming of distinct cell types (such as distinct T cell subsets, NK cells, and macrophages) to perform their specialized immune functions [190].

## Conclusions

The field of cellular immunotherapy has seen significant progress in recent years, with the initial clinical success of CAR T cell therapies in hematological malignancies leading to a surge of follow-up research. The focus has been on developing new gene engineering technologies for improved control over therapeutic cells after administration to the patient and overall product safety, efficacy, and flexibility. As is summarized in the respective sections of this review and Table 1, these technologies which include the use of safety switches and suicide genes, chemical and genetic ON- and OFF-switches, modulators of CAR gene expression levels, and adaptor universal immune receptor platforms, greatly expand the utility of CAR-modified immune cells for the potential patient population that could benefit from the therapy.

**Table 1:** Summary of described technologies and challenges they address

Category	CAR Control Technologies	Challenges Addressed	Mechanisms
1. Elimination of therapeutic cells	HSV-TK iC9 FADD CD20/Rituximab EGFRt/Cetuximab Cytosine deaminase Death signalobody, SFas Uridine metabolic switch	Safety	Incorporated genetic mechanism for on-demand cell elimination
2. Regulation of CAR gene expression	Tet-ON Res controlled CAR Zinc finger TF-based switch system LINTAD ReCoM Photothermal switch CAR FUS-CAR system HiCAR HypoxiCAR SynNotch logic CAR	Safety Efficacy Flexibility	Spatiotemporal control of CAR abundance limits off-tumor activity Prevent T cell exhaustion Controlling CAR expression location or using AND-logic provides access to more target antigens
3. Inducible CAR protein degradation	SWIFF-CAR Lenalidomide-induced CAR degradation PROTAC-mediated CAR degradation	Safety	Temporal regulation of CAR protein activity and abundance
4. Inducible formation of a functional CAR or inducible CAR disassembly	ON-switch split CAR DARRIC AvidCAR Lenalidomide ON switch split CAR Lipocalin-based ON-switch CAR FKBP/FRB-mcCAR LiCAR CRASH-IT switch CAR STOP-CAR Minocycline TetCAR CondCAR OFF-switch CAR	Safety Efficacy	Temporal regulation of CAR protein activity and abundance Prevent T cell exhaustion
5. Inhibitory CARs	iCAR Double-Arm CAR iKP (KIR2-PD1) CAR Tmod2 CAR NASCAR anti-HLA-DR iCAR	Safety Efficacy Flexibility	Limited off-tumor CAR activity Improved precision of antigen targeting More target antigens available (NOT-logic)
6. Modular universal adaptor CAR platforms	FcR-based ADCC CAR Anti-FITC CAR BBIR CAR mSA2 CAR AdCAR PNE specific CARs Convertible CAR Cp-Fab CAR SpyCatcher CAR SNAP-CAR UniCAR RevCAR SUPRA CAR bsCAR	Safety Efficacy Flexibility	Dose-dependent control of CAR abundance Simultaneous targeting of multiple antigens available Modular design for easy antigen retargeting

Category: corresponds to the respective review section. CAR Control Technologies: a list of technologies described in the section. Challenges Addressed: this column provides information about three challenges related to CAR cell control that were addressed with the described technologies. Mechanism: a short description of how each challenge was addressed using the described technologies

The ability to target multiple antigens with a single CAR T cell population allows for genetic cell programming to perform Boolean logic operations (e.g., AND, OR, NOT), and some new multicomponent technologies are being developed such as the colocalization-dependent protein CAR system (Co-LOCKR) [191]. In addition, researchers are exploring the possibility of coordinating a whole network of different CAR expression immune cell types tapping into their unique anti-cancer or regulatory roles [190].

Many of these advances were greatly facilitated by splitting the original CAR architecture into the soluble antigen recognition module and universal membrane receptor in various adaptor and universal immune receptor platforms [19, 20, 22]. Nevertheless, successful clinical translation of these novel technologies still depends on the same challenges, including the discovery and validation of reliable tumor antigens and detailed research of their biology (gene variants, expression regulation, trafficking, membrane shedding), and the development of high-affinity selective ligands.

Another aspect of cellular immunotherapy rapidly developing in parallel with advanced CAR technologies aims to produce superior and more affordable allogeneic “off-the-shelf” therapeutic cells enhanced by gene editing technologies [192–196]. The combination of various gene engineering techniques can modulate T cell activation and proliferation and have resistance to inhibitory signals from the tumor microenvironment. For example, when combined with CAR technology, knocking out immune checkpoint inhibitors, such as CTLA4, PD1 or PARP11[197], further improves the ability of CAR T cells to traffic to and infiltrate tumors, where overcoming the immunosuppressive microenvironment represents a prime challenge [198, 199]. Allogeneic T cells from healthy donors or derived from induced pluripotent stem cells (iPSC) were successfully generated, and demonstrated high preclinical efficacy [200, 201] and many clinical trials with allogenic CAR T cells are already underway (https://clinicaltrials.gov). In addition to conventional T cells, several other immune cell types, including NK cells and γδ T cells, are being successfully explored for cancer immunotherapy [202–205].

This review article aimed to comprehensively cover novel gene engineering technologies applied to control CAR T cells, and improve virtually all aspects of the original CAR design. Due to space limitations, the discussion could not include all studies and some other technologies, including the use of bispecific antibodies [206–208] and bispecific CARs such as Tandem CAR (TanCAR) with AND-logic function [209, 210]. All investigated modifications significantly improve tumor recognition and enhance killing efficacy and product safety while decreasing overall product cost. However, there is still much work to be done to overcome the challenges posed by the tumor microenvironment and to ensure that this promising therapeutic approach can be effectively translated into the clinic. For instance, careful consideration of possible immunogenicity issues posed by CAR components and other genetic modifications [211]. Cellular therapies, with the implementation of all currently developing improvements, can transform cellular immunotherapy and precision medicine fields, and become the preferred treatment option for various types of cancer and other diseases.

## Data Availability

Not applicable.

## Abbreviations

ADCC: Antibody-dependent cell-mediated cytotoxicity
AML: Acute myeloid leukemia
ASV: Asunaprevir
bsAb: Bispecific antibody
CID: Chemical inducer of dimerization
CRS: Cytokine release syndrome
CD: Cytosine deaminase
CDX: Cell line-derived xenografts
CAR: Chimeric antigen receptor
Dox: Doxycycline
EGFRt: Truncated human epidermal growth factor receptor
FADD: Fas-associated death domain-containing protein
Fab: Antigen binding fragment
FcR: Fc Receptor
FITC: Fluorescein isothiocyanate
FKBP: FK506-binding protein
FOLR1: Folate receptor 1
5-FC: 5-Fluorocytosine
5-FU: 5-Fluorouracil
GCV: Ganciclovir
GvHD: Graft-versus-host disease
Hsp: Heat-shock protein
HLA: Human leukocyte antigen
hRBP4: Human lipocalin retinol-binding protein 4
iC9: Inducible Caspase 9
ITAM: Immunoreceptor tyrosine-based activation motif
mAb: Monoclonal antibody
NK: Natural Killer cells
NLS: Nuclear localization signal
PNE: Peptide neoepitope
PROTAC: Proteolysis-targeting chimaera
Res: Resveratrol
scFv: Single-chain variable fragment
SMASh: Small molecule-assisted shutoff
SynNotch: Synthetic Notch
TCR: T cell receptor
TAA: Tumor-associated antigen
Tet: Tetracycline
UMPS: Uridine monophosphate synthetase
FUS: Focused ultrasound
HSV-TK: Herpes simplex virus thymidine kinase
